# Repeated administration of a flavonoid‐based formulated extract from citrus peels significantly reduces peripheral inflammation‐induced pain in the rat

**DOI:** 10.1002/fsn3.1566

**Published:** 2020-05-20

**Authors:** Tommaso Iannitti, Alessandro Di Cerbo, Anna Rita Loschi, Stefano Rea, Michiko Suzawa, Julio César Morales‐Medina

**Affiliations:** ^1^ Charles River Discovery Research Services UK Limited Portishead UK; ^2^ School of Biosciences and Veterinary Medicine University of Camerino Matelica Italy; ^3^ Miyauchi Citrus Research Center Shigoka‐Machi Takasaki Gunma Japan; ^4^ Centro de Investigación en Reproducción Animal CINVESTAV‐Universidad Autónoma de Tlaxcala Tlaxcala México

**Keywords:** citrus, flavonoid, forced swim test, inflammation, mechanical allodynia, open field

## Abstract

Depression‐related disorders are the first cause of disability worldwide according to the World Health Organization, and there is limited availability of effective antidepressant medications without side effects. Similarly, pain management is a public health concern particularly due to the increase in use of opioid medications, which have a significant side effect profile. Flavonoids can modulate numerous physiological functions including emotional and anti‐nociceptive processes. Gold lotion (GL) is a natural product based on the extract of six citrus peels rich in flavonoids (0.45 mg/ml) with numerous reported biological activities. In the present study, we investigated the effect of repeated administration of GL in a battery of behavioral tests, including the open field test (OFT), forced swim test (FST), and von Frey test (vFT), in rats. While the OFT measured anxiolytic‐related effects, the FST evaluated depression‐related behavior. The vFT evaluated mechanical allodynia in two rat models of peripheral inflammation induced by carrageenan or complete Freund's adjuvant (CFA) administration. Treatment with GL reduced mechanical allodynia after either carrageenan or CFA administration. On the other hand, repeated GL administration did not modulate any behavior evaluated by OFT or FST. Consumption of GL inhibits behavioral signs of inflammatory pain. Therefore, GL may be a valuable analgesic product to be used for inflammatory pain.

## Graphical abstract

Citrus extract has shown anti‐inflammatory and reducing inflammatory soup related molecules in vitro. In the present study, we show that the extract reduces mechanical allodynia.

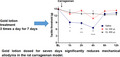

## 1 INTRODUCTION

Plants provide numerous metabolites including terpenoids, flavonoids, alkaloids, and phenolic compounds responsible for several health benefits (Flores, 2017; Sellami et al., 2018). Citrus peels have been extensively used in oriental traditional medicine (Hakim, Harris, & Ritenbaugh, 2000). They contain high levels of flavonoids, in particular, polymethoxyflavones (PMFs) with a wide range of biological activities (Li et al., 2006b, 2014; Li, Lo, & Ho, 2006a; Walle, 2007). Gold lotion (GL) is composed of peels of six citrus fruits found in Japan and was originally marketed for protection against ultraviolet radiation. In particular, GL reduced the expression of nitric oxide synthase (inducible form) and cyclooxygenases (1 and 2) in mouse colon (Lai et al., 2013). Moreover, GL reduced the expression of cytokines (interleukin 6 and 12), tumor necrosis factor α as well as the expression of major histocompatibility complex class I/II *in vitro*. All the aforementioned molecules are upregulated in the inflammatory soup (Basbaum et al., 2009) as well as in depression. Therefore, GL may display anti‐nociceptive and antidepressant‐related effects. Moreover, several *in vitro* and *in vivo* studies conducted with GL showed health benefits including anti‐cancer, anti‐inflammatory, and immunomodulatory properties (Lai et al., 2013; Li et al., 2014). GL contains a total measured content of at least 450 ppm flavonoids (0.45 mg/ml) and a PMF content of 106 ppm (0.1 mg/ml) (Lai et al., 2013). Moreover, since 2014, flavonoids have been used in 28% of studies looking at efficacy of new candidate therapies for neuropathic pain treatment (Quintans et al., 2014). In this study, we evaluated whether GL could have anti‐nociceptive and anti‐inflammatory properties in two well‐known *in vivo* models of peripheral inflammation and pain. The acute administration of carrageenan and complete Freund's adjuvant (CFA) has been extensively used to test potential analgesic and anti‐inflammatory pharmaceuticals (McCarson, 2015). For instance, the CFA model typically produces a chronic inflammatory response accompanied by mechanical allodynia lasting between 1 and 2 weeks (Morales‐Medina et al., 2017; Stein, Millan, & Herz, 1988). The carrageenan model is used to induce acute inflammatory pain and is accompanied by comparable behavioral features lasting for several hours (McCarson, 2015; Morales‐Medina et al., 2019).

Depression is a leading cause of disability worldwide (https://www.who.int/news-room/fact-sheets/detail/depression accessed on 23/02/2019) and presents high co‐morbidity with anxiety disorders. Given the limited efficacy of current antidepressants, the search for novel therapeutics is a major challenge in this field today. In this regard, flavonoids have shown antidepressant and anxiolytic properties (Song et al., 2017; Turkmenoglu et al., 2015). Hence, we evaluated whether GL could also present anxiolytic‐ and antidepressant‐related effects in rats. The forced swim test (FST) has been widely used to evaluate potential antidepressants (Lucki, 1997; Porsolt, Pichon, & Jalfre, 1977). In this test, large periods of immobility in the tank are interpreted as lack of motivation to escape, a trait observed in depression (Morales‐Medina et al., 2012a, 2012b). The open field test (OFT) measures anxiety‐related behavior based on the fact that rodents naturally avoid open bright spaces; the less crosses in the central part of the arena are interpreted as anxiogenic‐related behavior (Morales‐Medina et al., 2012a, 2012b). In summary, we analyzed whether repeated administration of GL could reduce mechanical allodynia in chronic and acute models of peripheral inflammatory pain and produce anxiolytic‐ and antidepressant‐related effects in rats.

## 2 MATERIALS AND METHODS

### 2.1 Study design

After repeated administration of GL, rats were divided in three groups. The first group received carrageenan, the second received CFA, and the third group performed two behavioral tests on two consecutive days, as previously reported (Morales‐Medina et al., 2013). The OFT was carried out on day 7, and the FST was performed on day 8. All behavioral tests were carried out during the light phase of the light–dark cycle (9:00–13:00). Rats were kept on a room with 12‐hr light/dark cycle, controlled temperature and humidity and *ad libitum* food and water. Eighteen rats per group were assessed.

### 2.2 Animals and housing

Male (*n* = 56) Wistar rats from 2 to 3 months of age (230–250 g) from our local animal facility at the Centro de Investigacion en Reproduccion Animal (CINVESTAV, Mexico) were housed two‐three per cage. The experimental unit is a single rat. All procedures complied with the National Institutes of Health Guide for the Care and Use of Laboratory Animals and ARRIVE guidelines for the use of animals (Kilkenny et al., 2010). Moreover, the procedures followed the technical guidelines for the production, care, and use of animals in the laboratory issued by SAGARPA Mexico (NOM‐062 ZOO‐1999) as well as a local committee. Each rat was only used once for each experiment. The experimental outcomes included behavioral data, and all efforts were made to minimize animal suffering. In case of signs of distress of the animals prior or during experimentation, the animals were assessed by a veterinarian and if necessary euthanized.

### 2.3 Gold lotion (GL)

GL was provided by Miyauchi Citrus Research Center, Ltd. (Japan), and stored at 4°C before the use according to the manufacturer's instructions (Lai et al., 2013; Li et al., 2014). GL is made of peels derived from six citrus fruits (navel oranges, *Citrus hassaku, Citrus limon, Citrus natsudaidai, Citrus miyauchi,* and *Satsuma*) with a total content of flavonoids equal to 0.45 mg/ml (Lai et al., 2013). Previous studies showed that flavonoids and PMFs concentrations in 10% GL were equivalent to 46.44 μg/ml and 10.61 μg/ml, respectively. From those flavonoids, the extract contained eight major chemical components: nobiletin (5.8 μg/ml), sinensetin (2.13 μg/ml), 3,5,6,7,8,3′,4′‐Hepta‐methoxyflavone (1.92 μg/ml), Tangeretin (1.06 μg/ml) 3,5,6,7,3′,4′‐Hexamethoxy‐flavone (0.31 μg/ml), 5,6,7,4′‐Tetramethoxy‐flavone (0.11 μg/ml), Naringin (25.36 μg/ml), and Hesperidin (10.47 μg/ml) (Lai et al., 2013). In all groups, GL was administered by oral gavage (randomized). Rats were administered with 200 µl GL (*n* = 6) or 400 µl GL (*n* = 6) or saline (200 µl; vehicle) (*n* = 6), 3 times daily for one week before behavioral testing. The sample size was calculated in base to previous experiments similar in nature. In all behavioral experiments, the observer was blinded to the treatment tested.

### 2.4 Behavioral testing

The experimental outcomes of the present study were behavioral changes in pain‐, depression‐, and anxiety‐related effects in the male rat.

#### 2.4.1 Von Frey test

Animals were acclimated to a stainless steel grid within individual Plexiglas boxes for 60 min and then tested for baseline mechanical allodynia using Von Frey (vF) monofilaments (Stoelting Inc., IL, USA) (Morales‐Medina et al., 2017, 2019; Rahn et al., 2014). For each animal, the mid‐plantar region of the left hind paw was stimulated with an incremental series of eight monofilaments of logarithmic stiffness. The 50% withdrawal threshold was determined using the up‐down method of Dixon, modified by Chaplan et al. (1994). First, an intermediate vF monofilament (number 4.31 exerting 2.0 g of force) was applied perpendicular to the plantar skin, causing a slight bending. If there was a positive response, consisting of a rapid withdrawal of the paw within 6 s, a smaller filament was applied. If there was a negative response, a larger filament was applied. Paw thickness was determined at baseline with a caliper according to the McCarson (2015).

#### 2.4.2 Complete Freund's adjuvant model

On day 4 after the initial treatment with GL, rats were administered CFA (Sigma, St. Louis, MO; 50 µl) to their ventral mid‐plantar left hind paw after baseline mechanical threshold testing. Mechanical responses and paw edema were measured on the left hind paw 2, 5 and 7 days later (Morales‐Medina et al., 2017).

#### 2.4.3 Carrageenan model

A 3% carrageenan solution (carrageenan from seaweed, Sigma, C1867‐5G) was prepared by dissolving carrageenan from seaweed in saline, heating to 37°C and vortexing. The solution was stored at 4°C overnight. One day after the last GL administration, rats received an intradermal injection of the carrageenan solution (3%; 50 µl) into their left hind paw. Mechanical responses and paw edema were assessed in the left hind paw starting at 1 hr postinjection. Afterwards, paw edema and mechanical responses were assessed at 2, 4, 6, and 12 hr.

#### 2.4.4 Open field test

This test was carried out in a square arena (90 × 90 × 45 cm) made of a black wooden box with a black floor and no top lid (Morales‐Medina et al., 2012b). The arena was divided into 64 equal‐size squares and received 300 LUX (Morales‐Medina et al., 2012b). The animals were recorded for a 10‐min period, and the behaviors evaluated were locomotion (horizontal behavior) and frequency of rearing and grooming (vertical behaviors) by an observer blind to the treatment. The testing apparatus was cleaned with ethanol solution (70%) after each trial. Behavior was determined anxiety‐related if the rat crosses less times the central part of the arena as well as enhanced rearing and grooming which is associated with stress coping behaviors (Kelly, Wrynn, & Leonard, 1997; Morales‐Medina et al., 2012b).

#### 2.4.5 Forced swim test

FST is commonly used to screen potential antidepressants (Lucki, 1997; Porsolt et al., 1977). In the present study, we used a modified version of the test where animals perform this task only once (Morales‐Medina et al., 2012a, 2012b). This behavioral test was performed in a white cylindrical tank (29 cm diameter and 43 cm height) with no top lid and was filled with water (25°C ± 2°C). Rats were placed in the swimming tank for a 10‐min period. A camera was mounted 1 m above the tank, and immobility was evaluated by an observer blind to the experimental conditions. Immobility time was recorded when the animal made minimum movements necessary to keep its body afloat. Increased time spent immobile to escape from water was determined as depression‐related behavior due to lack of motivation (Cryan & Mombereau, 2004). Following the test, rats were removed from the cylinder, cleaned with a towel, and placed under a red lamp until the fur was dry.

After the behavioral experiments, the rats were euthanized using a CO_2_ chamber and the bodies disposed accordingly.

### 2.5 Statistical analysis

Data were analyzed using GraphPad Prism (GraphPad Software Inc., San Diego, CA, USA). Mechanical threshold and paw edema data were analyzed using a two‐way analysis of variance (ANOVA) followed by Dunnett's *post hoc* test for multiple comparisons. Immobile, struggling, swimming, total locomotion, central locomotion, and rearing and grooming data were analyzed using a one‐way ANOVA with Dunnett's *post hoc* test for multiple comparisons.

## 3 RESULTS

A comprehensive battery of behavioral tests was used to evaluate GL anti‐inflammatory, anti‐nociceptive, antidepressant‐related, and anxiolytic‐like effects. Horizontal and vertical activities were evaluated in the OF apparatus, immobility in the FST, mechanical allodynia and paw edema using the Von Frey test and a caliper, respectively, in rats administered with CFA or Carrageenan. No data were excluded from the present study, and no treatment‐related adverse effects such as loss or gain of body weight, diarrhea, or shivering were observed in the animals tested.

### 3.1 Effect of repeated administration of GL in rats administered with Carrageenan

**FIGURE 1:**
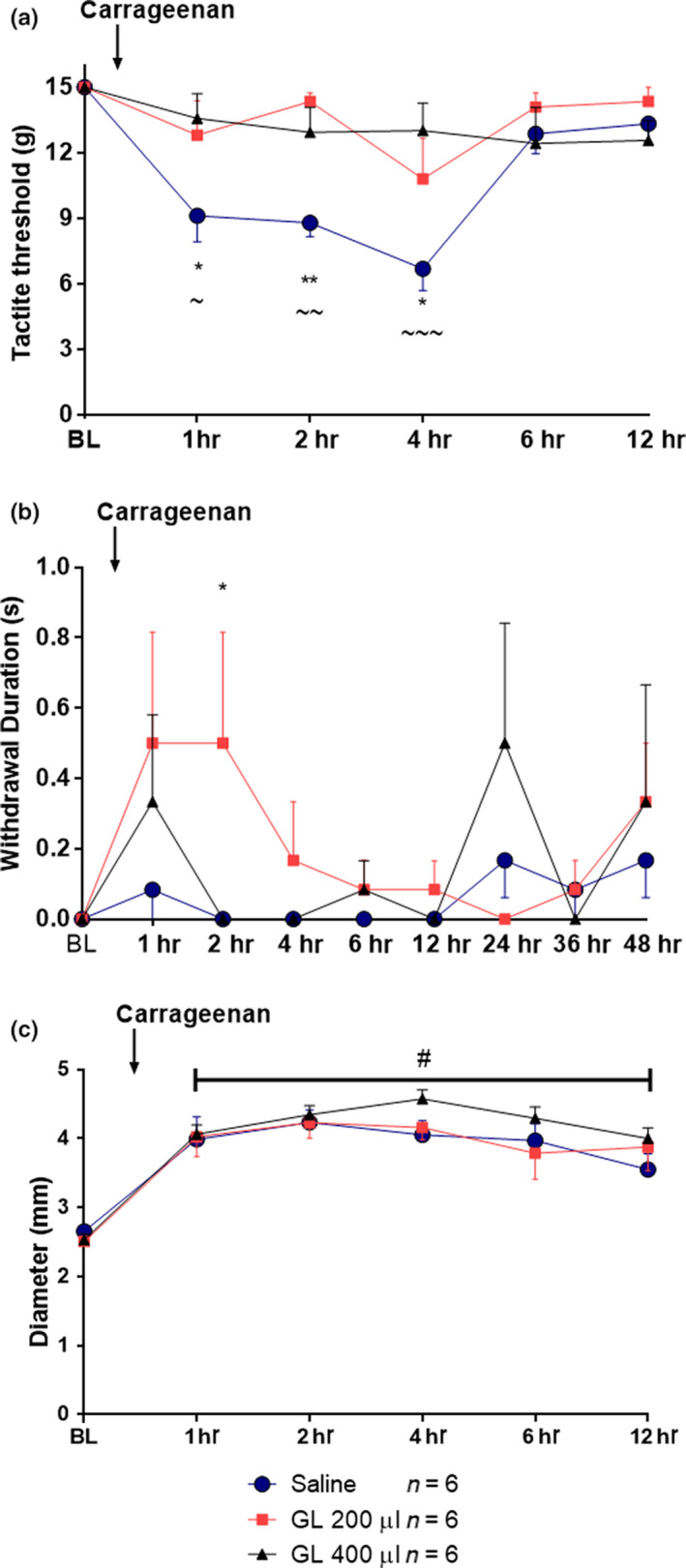
Time course of the effect of gold lotion postcarrageenan administration on mechanical hypersensitivity and paw edema in male rats. (a) shows mechanical response thresholds at baseline (BL; Day 0) and at 1, 2, 4, 6, and 12 hr postcarrageenan administration. (b) represents cold hypersensitivity postcarrageenan administration. (c) shows paw edema. * and ~ indicate *p* < .05 for GL 200 µl and GL 400 µl versus saline, respectively, ** and ~~ indicate *p* < .01 for GL 200 µl and GL 400 µl versus saline, respectively; ~~~ indicates *p* < .001 for GL 400 µl versus saline; # represents *p* < .05 compared with baseline. Arrow represents the time of carrageenan injection. Data are presented as mean ± *SEM*, *n* = 6 for all groups

We observed a significant increase in mechanical thresholds following administration of GL [time *F*(8, 134) = 5.301, *p* = .0183; Treatment *F*(2, 134) = 4.12, *p* < .001; Interaction *F*(16, 134) = 2.757, *p* = 2.757] with 200 μl at 1, 2, and 4 hr compared with saline (*p* < .05, *p* < .01, and *p* < .05, respectively; Figure 1a].

We also evaluated the effect of GL on thermal cold allodynia (Figure 1b). No change in paw diameter was observed at all the GL doses tested compared with saline (Figure 1c).

### 3.2 Effect of repeated administration of GL in rats administered with CFA

**FIGURE 2:**
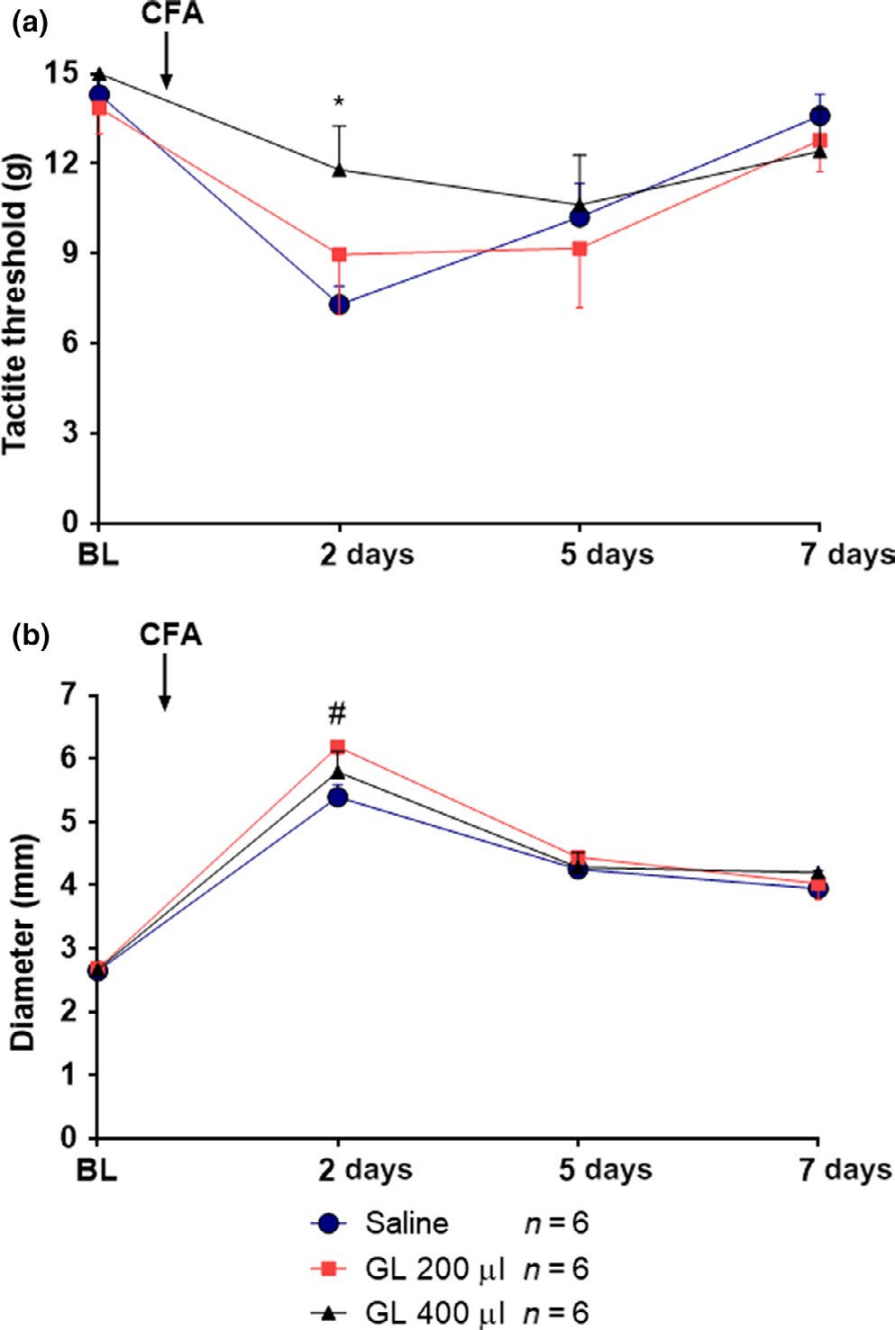
Time course of the effect of gold lotion administration postcomplete Freund adjuvant (CFA) administration on mechanical hypersensitivity and paw edema in male rats. (a) shows mechanical response thresholds at baseline (BL; Day 0) and at 2, 5, and 7 days post‐CFA administration. (b) shows paw edema. Arrow represents the time of CFA injection. * indicates *p* < .05 for GL 400 μl versus saline, # indicates *p* < .05 for all groups compared with BL. Data are presented as mean ± *SEM*, *n* = 6 for all groups

On day 2 after CFA administration, GL 400 μl reduced the mechanical hypersensitivity induced by CFA administration [time *F*(3, 68) = 12.01, *p* < .001; Treatment *F*(2, 68) = 1.361, *p* = .29; Interaction *F*(6, 68) = 1.108, *p* = .3669; *p* < .05; Figure 2a].

CFA increased the paw edema at day 2 compared to baseline (*p* < .05) (Figure 2b).

### 3.3 Effect of repeated administration of GL in the OFT

**FIGURE 3:**
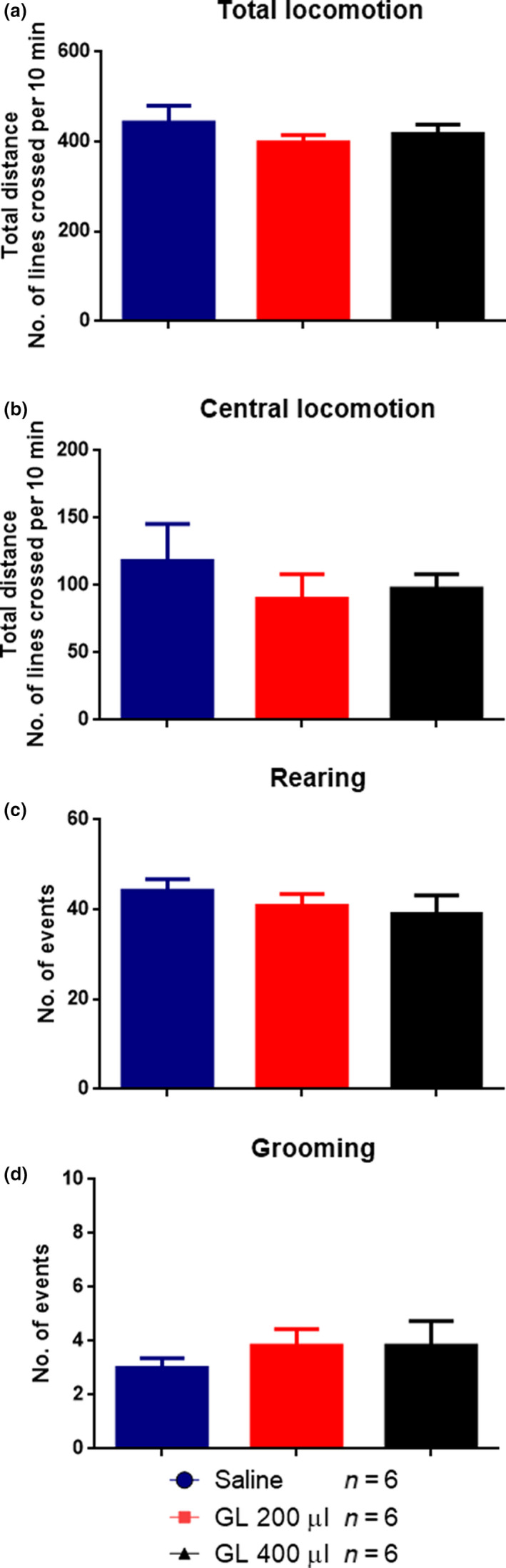
Effect of repeated administration of gold lotion in vertical and horizontal behaviors in the open field test in male rats. Gold lotion fails to modulate the local locomotion (a), central locomotion (b), rearing (c), or grooming (d). Data are presented as mean ± *SEM*, *n* = 6 for all groups

In the OFT, the administration of GL did not modulate locomotion (central or total), rearing, or grooming (Figure 3a–d).

### 3.4 Effect of repeated administration of GL in the FST

**FIGURE 4:**
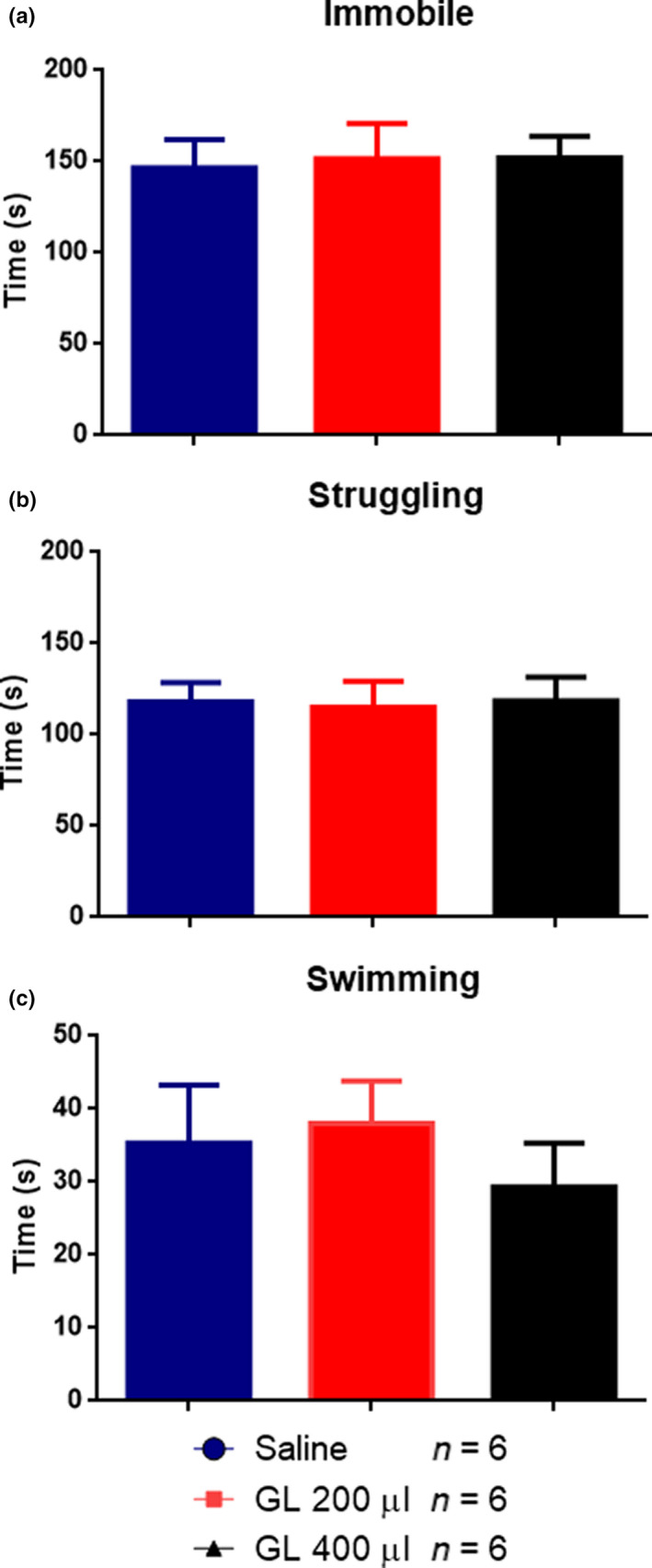
Effect of repeated administration of gold lotion in active and inactive behaviors in the forced swim test (FST) in male rats. Gold lotion fails to modulate the immobile (a), struggling (b), or swimming (c) component of the FST. Data are presented as mean ± *SEM*, *n* = 6 for all groups

In the FST, the repeated administration of GL did not modify any of the behavioral parameters evaluated (time spent immobile, struggling, or swimming (Figure 4a–c).

## 4 DISCUSSION

In the present study, the peel extract of six fruits (GL) was evaluated for its possible anti‐nociceptive, anti‐inflammatory, anxiolytic, and antidepressant activity in well‐characterized behavioral assays. One limitation of the present study is that was carried out only in male rats, therefore not allowing comparison between sexes.

The carrageenan model has been extensively used to study acute peripheral pain and inflammation (Iannitti, Graham, & Dolan, 2015; Morales‐Medina et al., 2019). In this model, behavioral hypersensitivity is well established at 1 hr and peaks at 4 hr postcarrageenan, with the edema being present already at 1 hr postcarrageenan administration. Carrageenan produces effects locally, systemically, and at the spinal cord levels. Indeed, the edema induced locally by carrageenan administration is the result of the upregulation of several inflammatory mediators including prostaglandins (Vinegar, Schreiber, & Hugo, 1969), nitric oxide released from polymorphonuclear leukocytes at the site of inflammation (Ialenti et al., 1992), and TNF‐α (Sekut et al., 1994). Transient receptor potential vanilloid 1 (TRPV1) is key in carrageenan‐induced inflammatory pain, as vanilloid receptor null mice do not develop carrageenan‐induced thermal hyperalgesia (Davis et al., 2000). Moreover, carrageenan‐induced inflammation is also associated with systemic release of cytokines. For example, carrageenan administration into the hind paw induces an increase in cytokine‐induced neutrophil chemoattractant 1 in plasma 6 hr postinjection (Loram et al., 2007). A systemic effect of carrageenan is also confirmed by a threefold increase in serum interferon‐gamma at 72 hr and a twofold increase in serum IL‐1β at 48 hr, after injection into the hind paw (Huber et al., 2006). Plasma concentration of leptin is also increased following carrageenan‐induced rat paw edema (Gualillo et al., 2000). In the spinal cord, following carrageenan administration, COX‐2 mRNA is induced bilaterally (Ichitani et al., 1997), and prostaglandins are released (Baba et al., 2001; Yaksh et al., 2001) resulting in central sensitization with a subsequent release of substance P (Honor et al., 1999). The aim of this study was to determine GL efficacy but we did not perform any pharmacodynamics readouts in the carrageenan model. Therefore, further studies will have to determine whether GL exerts its effects locally or in the central nervous system and what mechanisms are responsible.

The CFA rodent model is used routinely to study the efficacy of novel analgesics and anti‐inflammatory treatments. CFA administration results in a chronic inflammatory response, which is accompanied by increased sensitivity to thermal and mechanical stimuli, and reduced weight bearing, lasting between 1 and 2 weeks (McCarson, 2015; Stein et al., 1988). Indeed, CFA induces the expression of COX‐2 and prostaglandin E_2_ in the spinal cord within hours (Fang et al., 2013). While the time course of mechanical allodynia and inflammation is different, both CFA and carrageenan induce a similar profile of molecules of the inflammatory soup. In this study, we assessed GL efficacy in the CFA model but it was beyond the aim of this study to determine its mechanism of action.

GL (200 μl) administered orally reduced the expression of COX‐2 and iNOS in azoxymethane‐induced colonic tumorigenesis tissues of mice (Lai et al., 2013). Moreover, GL reduced the expression of TNF‐α, interleukin (IL)‐6, and IL‐12 in cell cultures of mouse bone marrow‐derived challenged with lipopolysaccharide (LPS) (Li et al., 2014). Henceforth, the known mechanisms of GL reducing the aforementioned markers of inflammation and pain may mediate, at least partially, the anti‐nociceptive effects of GL observed in the present study. For example, it is known that intracerebroventricular administration per se induce thermal hyperalgesia in rats (Oka et al., 1995). GL may produce its anti‐nociceptive effects, at least partially, by reducing the levels of COX‐2, iNOS, TNF‐α, and certain interleukins.

The present results show that repeated administration with GL do not modify the behavior in the FST or OF in rats. Recently, standardized *Citrus unshiu* peel extract reduced the immobility in the FST as well as the tail suspension test (TST) (a mice analog of the FST) without modifying the behavior in the OFT in dexamethasone‐induced depression‐like behavior in mice (Lim et al., 2018). Moreover, extract with *Citrus aurantium* L. decreased anxiety‐related behaviors in mice (Pultrini Ade et al., 2006). It is important to note that the extracts with *Citrus unshiu* and *Citrus aurantium* present a different pharmaceutical composition from GL, which could explain the lack of effects observed in this study.

## 5 CONCLUSION

The present study shows that GL has anti‐nociceptive and anti‐inflammatory properties but does not show anxiolytic or antidepressive effects.

## FUNDING INFORMATION

This work was supported by a Miyauchi Citrus Research Center research grant given to TI, ADC and JCMM.

## AUTHORS' CONTRIBUTIONS

TI, ADC, JCMM, and MS conceived of the presented idea. TI and JCMM performed the animal experiments. ARL and SR verified the analytical methods and supervised the findings of this work. All authors discussed the results and contributed to the final manuscript.
